# Crystal structure and Hirshfeld surface analysis of ethyl (3*E*)-5-(4-chloro­phen­yl)-3-{[(4-chloro­phen­yl)formamido]­imino}-7-methyl-2*H*,3*H*,5*H*-[1,3]thia­zolo[3,2-*a*]pyrimidine-6-carboxyl­ate

**DOI:** 10.1107/S205698902200603X

**Published:** 2022-07-26

**Authors:** Shaaban K. Mohamed, Joel T. Mague, Mehmet Akkurt, Abdallah M. Alfayomy, Sahar M. Abou Seri, Shaban A. A. Abdel-Raheem, Mokhtar A. Abd Ul-Malik

**Affiliations:** a Chemistry and Environmental Division, Manchester Metropolitan University, Manchester, M1 5GD, England; b Chemistry Department, Faculty of Science, Minia University, 61519 El-Minia, Egypt; c Department of Chemistry, Tulane University, New Orleans, LA 70118, USA; d Department of Physics, Faculty of Sciences, Erciyes University, 38039 Kayseri, Turkey; e Department of Pharmaceutical Chemistry, Faculty of Pharmacy, Al-Azhar University, Assiut, 71524, Egypt; f Department of Pharmaceutical Chemistry, Faculty of Pharmacy, Cairo University, Kasr El-Aini Street, Cairo, PO Box, 11562, Egypt; g Soil, Water, and Environment Research Institute, Agricultural Research Center, Giza, Egypt; h Chemistry Department, Faculty of Applied Science, Taiz University, Taiz, Yemen; Katholieke Universiteit Leuven, Belgium

**Keywords:** crystal structure, hydrogen bond, thia­zole, pyrimidine, Hirshfeld surface analysis

## Abstract

In the title mol­ecule, C_23_H_20_Cl_2_N_4_O_3_S, the thia­zole ring is planar while the pyrimidine unit fused to it adopts a screw-boat conformation. In the crystal, thick sheets parallel to the *bc* plane are formed by N—H⋯N, C—H⋯N and C—H⋯O hydrogen bonds together with π–π inter­actions between the formamido carbonyl groups and the thia­zole rings. Hirshfeld surface analysis indicates that the most important contributions to the crystal packing are from H⋯H (30.9%), Cl⋯H/H⋯Cl (20.7%), C⋯H/H⋯C (16.8%) and O⋯H/H⋯O (11.4%) inter­actions.

## Abstract 2

The thia­zole ring is planar while the pyrimidine unit fused to it adopts a screw-boat conformation In the crystal, N—H⋯N plus C—H⋯N hydrogen bonds form helical chains along the *b*-axis direction, which are linked into thick sheets parallel to the *bc* plane by C—H⋯O hydrogen bonds and π–π inter­actions between the formamido carbonyl groups and the thia­zole rings.

## 1. Chemical context

Several compounds bearing 1,3,4-oxa­diazole have been reported to exhibit significant anti­cancer activities (Yadagiri *et al.*, 2015 ▸; Valente *et al.*, 2014 ▸; El-Din *et al.*, 2015 ▸). On the other hand, pyrimidine-based compounds have shown significant activity against cancer and tumor cells (Tolba *et al.*, 2022 ▸). Compounds combining the pharmacophores di­hydro­pyrimidine and 1,3,4-oxa­diazole have been prepared with the aim of developing potent anti­cancer agents (Ragab *et al.*, 2017 ▸). The target hybrids have been synthesized through condensation of 6-methyl-4-aryl-1,2,3,4-tetra­hydro­pyrimidine-2(1*H*)-thione derivatives and 2-(chloro­meth­yl)-5-aryl-1,3,4-oxa­diazole derivatives and screened for their *in vitro* cytotoxic activity against 60 cancer cell lines according to NCI (USA) protocols (Skehan *et al.*, 1990 ▸). Unexpectedly, an intra­molecular cyclization and ring opening of 1,3,4-oxa­diazole has occurred and the title compound was chosen as an example of this series for further structural elucidation through X-ray crystallography. 
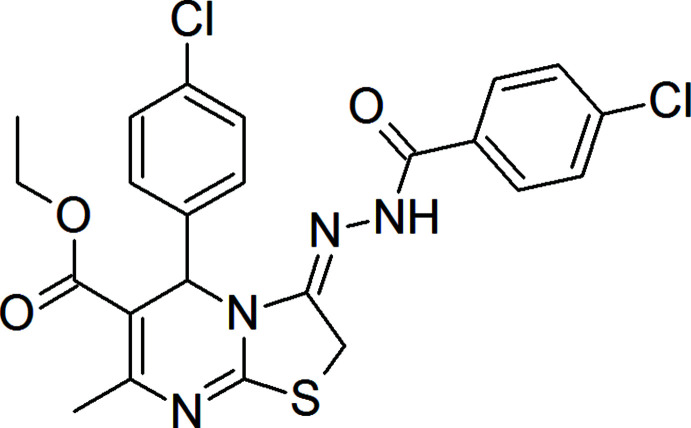


## 2. Structural commentary

**Figure 1:**
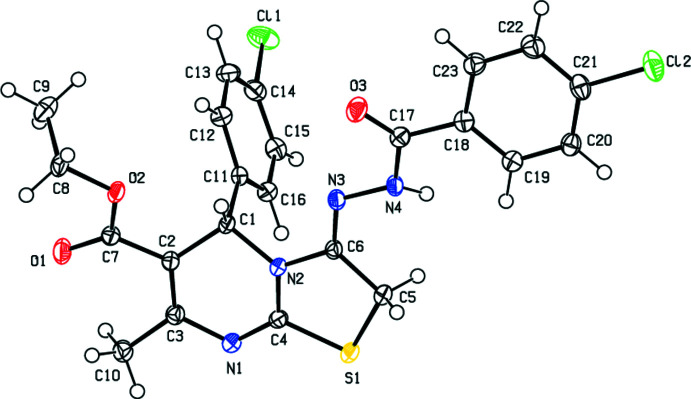
The title mol­ecule with the labelling scheme and 50% probability ellipsoids.

In the title compound, (Fig. 1 ▸), the thia­zole ring is planar (r.m.s. deviation of the fitted atoms = 0.001 Å) and the C11–C16 and C18–C23 benzene rings are inclined to it by 88.95 (8) and 11.47 (7)°, respectively. The pyrimidine ring (C1/C2/C3/N1/C4/N2) exhibits a screw-boat conformation with puckering parameters (Cremer & Pople, 1975 ▸) of *Q*(2) = 0.2383 (15) Å and φ(2) = 188.4 (4)°. This ring is folded about the C1⋯N1 axis by 19.9 (1)°. The torsion angles about the bonds of the *N*′-methyl­ideneformohydrazide link between the chloro­phenyl ring and the 2,3-di­hydro-5*H*-[1,3]thia­zolo[3,2-*a*]pyrimidine ring system are: N2—C6=N3—N4 = −177.82 (12)°, C6=N3—N4—C17 = −171.54 (13)° and N3—N4—C17—C18 = −175.14 (12)°. The stereochemistry about the imine function C6=N3 is *E*.

## 3. Supra­molecular features and Hirshfeld surface analysis

**Figure 2:**
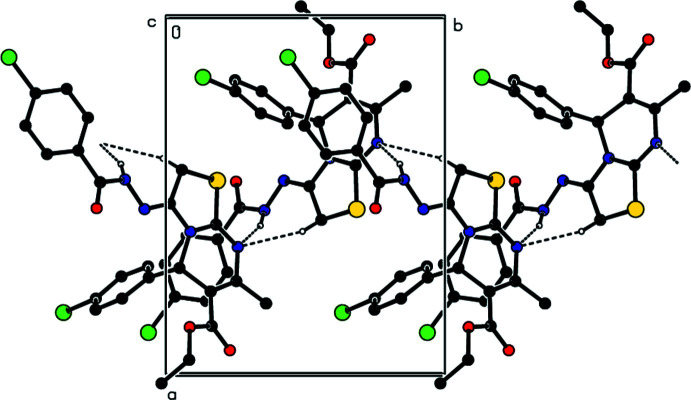
A portion of the hydrogen-bonded chain viewed along the *c*-axis direction. N—H⋯N and C—H⋯N hydrogen bonds are shown. H atoms not involved in these inter­actions have been omitted for clarity.

**Figure 3:**
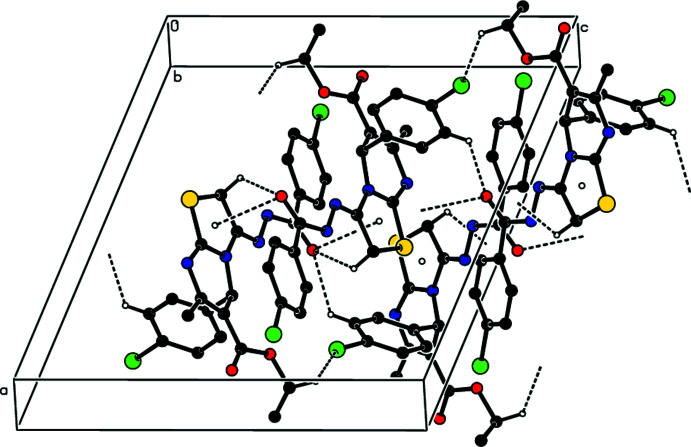
Detail of the C—H⋯O and C—H⋯Cl hydrogen bonds and the π-inter­actions down the *b*-axis. H atoms not involved in these inter­actions have been omitted for clarity.

**Figure 4:**
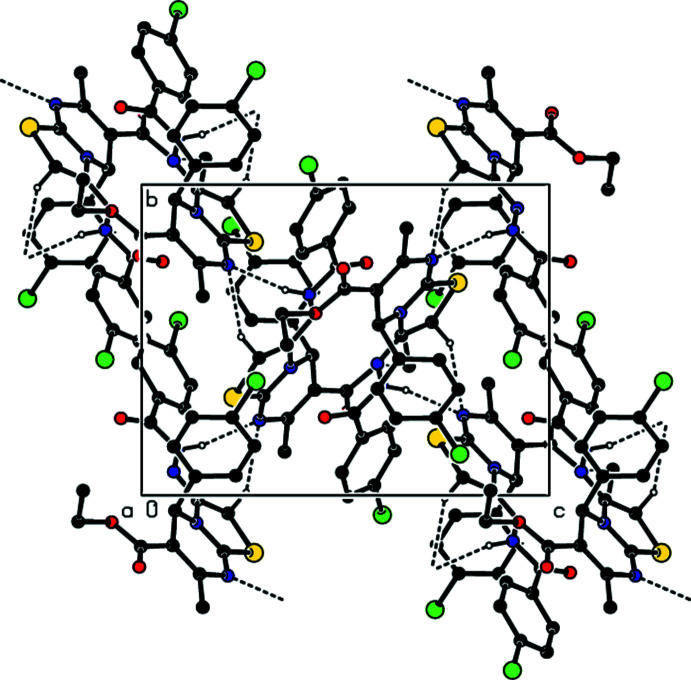
Packing viewed along the *a*-axis direction with inter­molecular inter­actions shown as in Fig. 2 ▸.

In the crystal, a combination of N4—H4⋯N1 and C5—H5*B*⋯N1 hydrogen bonds (Table 1 ▸) form helical chains extending along the *b*-axis direction (Fig. 2 ▸). The chains are connected by C5—H5*A*⋯O3, C15—H15⋯O3 and C8—H8*B*⋯Cl1 hydrogen bonds as well as centrosymmetrically related π-inter­actions between the C17=O3 carbonyl groups and the thia­zole rings [O3⋯*Cg*1^i^ = 3.0299 (14) Å, C17⋯*Cg*1^i^ = 3.4656 (16) Å, C17=O3⋯*Cg*1^i^ = 100.48 (10)°; Table 2 ▸ and Fig. 3 ▸; *Cg*1 is the centroid of the thia­zole ring, symmetry code: (i) 1 − *x*, 1 − *y*, 1 − *z*] into thick layers parallel to the *bc* plane (Fig. 4 ▸).

**Figure 5:**
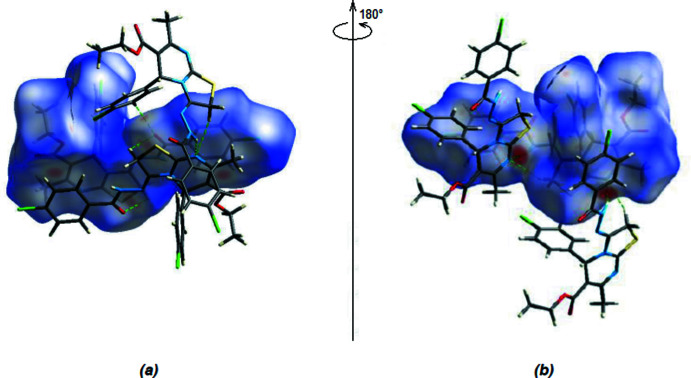
(*a*) Front view and (*b*) back view of the three-dimensional Hirshfeld surface of the title compound plotted over *d*_norm_ in the range −0.4486 to +1.3171 a.u.

**Figure 6:**
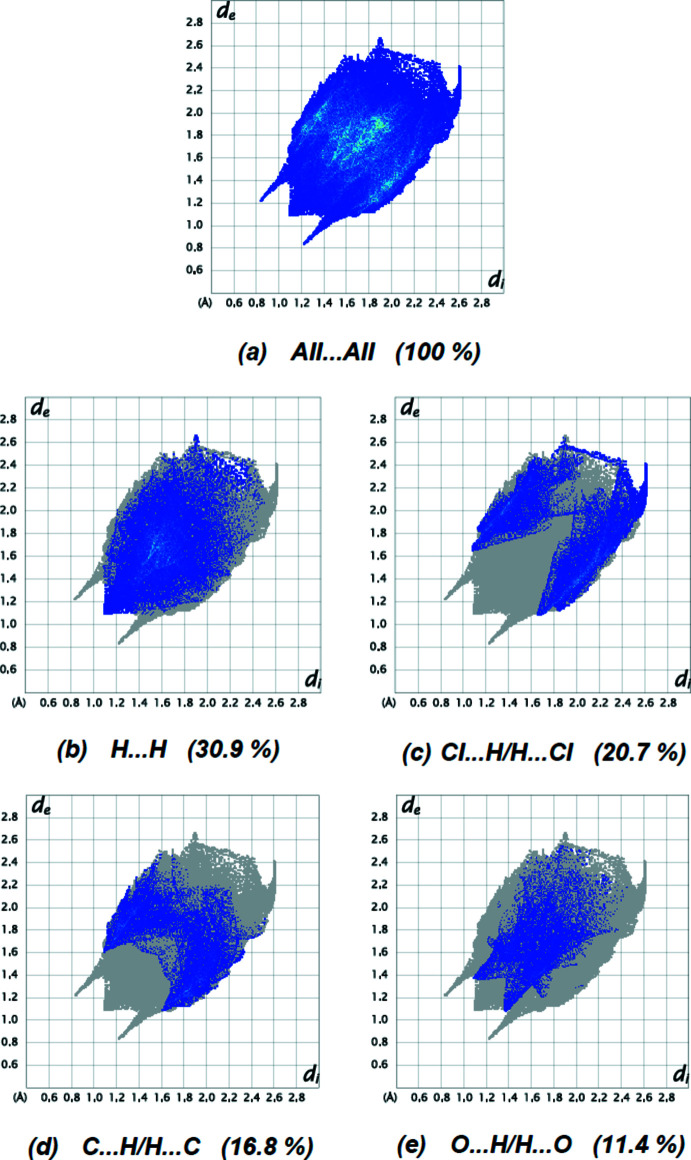
Two-dimensional fingerprint plots for the title compound, showing (*a*) all inter­actions, and delineated into (*b*) H⋯H, (*c*) Cl⋯H/H⋯Cl, (*d*) C⋯H/H⋯C and (*e*) O⋯H/H⋯O inter­actions. The *d*_i_ and *d*_e_ values are the closest inter­nal and external distances (in Å) from given points on the Hirshfeld surface.

A Hirshfeld surface analysis was performed using *Crystal Explorer 17.5* (Turner *et al.*, 2017 ▸) to visualize the inter­molecular inter­actions. The Hirshfeld surface mapped over *d*_norm_ (Fig. 5 ▸) shows the expected bright-red spots near atoms N1, O3, H5*A*, H5*B* and H15 involved in the C—H⋯O and C—H⋯N hydrogen-bonding inter­actions (Table 1 ▸) and short contacts (Table 2 ▸). Analysis of the two-dimensional fingerprint plots (Fig. 6 ▸) reveals that H⋯H (30.9%), Cl⋯H/H⋯Cl (20.7%), C⋯H/H⋯C (16.8%) and O⋯H/H⋯O (11.4%) inter­actions make the greatest contributions to the surface contacts. The remaining contributions for the title compound are from N⋯H/H⋯N, S⋯H/H⋯S, S⋯C/C⋯S, N⋯C/C⋯N, S⋯N/N⋯S, C⋯C, Cl⋯O/O⋯Cl, O⋯C/C⋯O, N⋯N, Cl⋯Cl, S⋯O/O⋯S, O⋯N/N⋯O and Cl⋯C/C⋯Cl contacts, which are each less than 4.5% and have a negligible effect on the packing. The percentage contributions of all inter­actions are given in Table 3 ▸.

## 4. Database survey

A search of the Cambridge Structural Database (CSD Version 5.39; Groom *et al.*, 2016 ▸) for similar structures with the 2,3-di­hydro-5*H*-[1,3]thia­zolo[3,2-*a*]pyrimidine ring system showed the three closest are those of *rac*-(2′′S*,2′R*,4′R*,5′′R*,)-ethyl 4′-meth­oxy­carbonyl-5′′-(4-meth­oxy­phen­yl)-1′,7′′-dimethyl-2,3′′-dioxo-2′′,3′′-di­hydro­indoline-3-spiro-2′-pyrrolidine-3′-spiro-2′′-thia­zolo[3,2-*a*]pyrimidine-6′′-carboxyl­ate [CSD refcode PONWUL (**I**); Hou *et al.*, 2009 ▸], 3-(4-fluoro­phen­yl)-2-sulfanyl­idene-5-(tri­fluoro­meth­yl)-2,3-di­hydro­[1,3]thia­zolo[4,5-*d*]pyrimidin-7(6*H*)-one toluene solvate [WEGSUA (**II**); Becan *et al.*, 2022 ▸] and 7-ethyl­amino-3-phenyl-5-(tri­fluoro­meth­yl)[1,3]thia­zolo[4,5-*d*]pyrimidine-2(3*H*)-thione [WEG­TAH (**III**); Becan *et al.*, 2022 ▸].

In compound (**I**), which crystallizes in the triclinic space group *P* 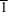 , the two spiro junctions link a planar 2-oxindole ring [with a mean deviation from the plane of 0.0319 (3) Å], a pyrrolidine ring in an envelope conformation and a thia­zolo[3,2-*a*]pyrimidine system. Two mol­ecules are connected into a dimer by two N—H⋯O hydrogen bonds, forming an 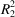 (8) graph-set motif.

Compound (**II**) crystallizes as a hemi-solvate in the triclinic space group *P* 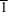 . The asymmetric unit is composed of one mol­ecule in the lactim form and half of a toluene mol­ecule. In the crystal structure of (**II**), the mol­ecules are linked into a centrosymmetric dimer by N—H⋯O hydrogen bonds. Such dimers are further linked *via* rather weak C—H⋯S and C—H⋯F inter­actions. In addition, aromatic π–π stacking inter­actions are also observed.

Compound (**III**) crystallizes in the *P*2_1_/*n* space group with one mol­ecule in the asymmetric unit. Both the thia­zolo­pyrimidine and the phenyl rings are flat and subtend a dihedral angle of 70.8 (1)° to each other. In the crystal of (**III**), N—H⋯S hydrogen bonds link the mol­ecules into zigzag chains running along the *b*-axis direction. The inter­chain contacts are provided by weak C—H⋯S and C—H⋯F bonds while C—H⋯π and π–π inter­actions generate the three-dimensional network.

## 5. Synthesis and crystallization

A mixture of ethyl 4-(4-chloro­phen­yl)-6-methyl-2-thioxo-1,2,3,4-tetra­hydro­pyrimidine-5-carboxyl­ate (2 mmol), 2-(chloro­meth­yl)-5-(4-chloro­phen­yl)-1,3,4-oxa­diazole (2 mmol), potassium iodide (2 mmol) and triethyl amine (2.5 mmol), was refluxed for 4h in absolute ethanol (20 mL). The reaction mixture was poured onto crushed ice (40 g) and acidified with acetic acid (2 mL). The deposited precipitate was filtered off, washed with cold water, dried and crystallized from a methanol/DMF mixture 4:1 (*v*/*v*).

Yield: 80%; melting point: 477–779 K; IR (KBr, ν_max_/cm^−1^) : 3402, 3174, 1708, 1693, 1651.^1^H NMR (400 MHz, DMSO-*d*_6_) δ 10.82 (*s*, 1H, NH), 7.85 (*d*, *J* = 8.3 Hz, 2H, Ar—H), 7.57 (*d*, *J* = 8.4 Hz, 2H, Ar—H), 7.41 (*dd*, *J* = 8.8, 8.4 Hz, 4H, Ar—H), 6.10 (*s*, 1H, C4—H), 4.46 (*d*, *J* = 17.4 Hz, 1H, S—CH_2_), 4.36 (*d*, *J* = 17.4 Hz, 1H, S—CH_2_), 4.03 (*q*, *J* = 5.2 Hz, 2H, CH_2_—CH_3_), 2.34 (*s*, 3H, C6—CH_3_), 1.12 (*t*, *J* = 7.1 Hz, 3H, CH_2_—CH_3_). ^13^C NMR (100 MHz, DMSO-*d*_6_) δ 165.02, 162.17, 153.72, 153.44, 139.52, 136.36, 132.78, 132.16, 129.82, 129.55, 128.41, 128.30, 105.37, 59.85, 54.69, 28.11, 22.66, 13.97. Analysis calculated for C_23_H_20_Cl_2_N_4_O_3_S (503.40): C 54.88, H 4.00, N 11.13. Found: C 55.13, H 3.94, N 11.36.

## 6. Refinement details

Crystal data, data collection and structure refinement details are summarized in Table 4 ▸. Only the hydrogen atoms of the methyl group attached to C10 were included as riding contributions in idealized positions since independent refinement of them led to an unsatisfactory geometry for this methyl group. All the remaining C and N-bound hydrogen atoms were found in difference-Fourier maps and they were refined freely.

## Supplementary Material

Crystal structure: contains datablock(s) I, global. DOI: 10.1107/S205698902200603X/vm2265sup1.cif

Structure factors: contains datablock(s) I. DOI: 10.1107/S205698902200603X/vm2265Isup2.hkl

Supporting information file. DOI: 10.1107/S205698902200603X/vm2265Isup3.cml

CCDC reference: 2177430

Additional supporting information:  crystallographic information; 3D view; checkCIF report

## Figures and Tables

**Table 1:** Hydrogen-bond geometry (Å, °)

*D*—H⋯*A*	*D*—H	H⋯*A*	*D*⋯*A*	*D*—H⋯*A*
N4—H4⋯N1^i^	0.89 (2)	2.16 (2)	3.0076 (18)	158 (2)
C5—H5*A*⋯O3^ii^	0.98 (2)	2.533 (19)	3.081 (2)	115.3 (14)
C5—H5*B*⋯N1^i^	0.98 (2)	2.57 (2)	3.453 (2)	150.1 (16)
C8—H8*B*⋯Cl1^iii^	1.02 (2)	2.77 (2)	3.4430 (17)	123.1 (14)
C15—H15⋯O3^iv^	0.95 (2)	2.54 (2)	3.1682 (19)	124.5 (17)

Symmetry codes: (i) 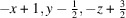 ; (ii) 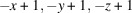 ; (iii) 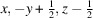 ; (iv) 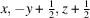 .

**Table 2:** Summary of short inter­atomic contacts (Å) in the title compound

Contact	Distance	Symmetry operation
Cl1⋯H10*B*	2.96	*x*, −1 + *y*, *z*
H4⋯N1	2.16	1 − *x*, − 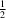 + *y*, 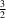 − *z*
H15⋯O3	2.54	*x*, 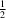 − *y*, 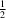 + *z*
H13⋯Cl2	2.91	1 − *x*, −*y*, 1 − *z*
H5*A*⋯O3	2.53	1 − *x*, 1 − *y*, 1 − *z*
H20⋯H9*B*	2.53	1 + *x*, 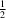 − *y*, 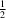 + *z*
H9*A*⋯H9*A*	2.43	−*x*, 1 − *y*, 1 − *z*

**Table 3:** Percentage contributions of inter­atomic contacts to the Hirshfeld surface for the title compound

Contact	Percentage contribution
H⋯H	30.9
Cl⋯H/H⋯Cl	20.7
C⋯H/H⋯C	16.8
O⋯H/H⋯O	11.4
N⋯H/H⋯N	4.5
S⋯H/H⋯S	3.4
S⋯C/C⋯S	2.9
N⋯C/C⋯N	1.4
S⋯N/N⋯S	1.4
C⋯C	2.8
Cl⋯O/O⋯Cl	0.9
O⋯C/C⋯O	0.9
N⋯N	0.8
Cl⋯Cl	0.4
S⋯O/O⋯S	0.3
O⋯N/N⋯O	0.2
Cl⋯C/C⋯Cl	0.1

**Table 4:** Experimental details

Crystal data	
Chemical formula	C_23_H_20_Cl_2_N_4_O_3_S
*M*_r_	503.39
Crystal system, space group	Monoclinic, *P*2_1_/*c*
Temperature (K)	150
*a*, *b*, *c* (Å)	14.8117 (18), 10.7086 (13), 15.1887 (19)
β (°)	112.417 (3)
*V* (Å^3^)	2227.1 (5)
*Z*	4
Radiation type	Cu *K*α
μ (mm^−1^)	3.80
Crystal size (mm)	0.21 × 0.18 × 0.08

Data collection	
Diffractometer	Bruker D8 VENTURE PHOTON 100 CMOS
Absorption correction	Numerical (*SADABS*; Krause *et al.*, 2015 ▸)
*T*_min_, *T*_max_	0.59, 0.76
No. of measured, independent and observed [*I* > 2σ(*I*)] reflections	16958, 4497, 4000
*R*_int_	0.031
(sin θ/λ)_max_ (Å^−1^)	0.625

Refinement	
*R*[*F* ^2^ > 2σ(*F* ^2^)], *wR*(*F* ^2^), *S*	0.032, 0.082, 1.05
No. of reflections	4497
No. of parameters	367
H-atom treatment	H atoms treated by a mixture of independent and constrained refinement
Δρ_max_, Δρ_min_ (e Å^−3^)	0.23, −0.35

Computer programs: *APEX3* and *SAINT* (Bruker, 2016 ▸), *SHELXT* (Sheldrick, 2015*a* ▸), *SHELXL2018/1* (Sheldrick, 2015*b* ▸), *ORTEP-3 for Windows* (Farrugia, 2012 ▸) and *PLATON* (Spek, 2020 ▸).

## supplementary crystallographic information

### Crystal data

**Table:**

C_23_H_20_Cl_2_N_4_O_3_S	*F*(000) = 1040
*M**_r_* = 503.39	*D*_x_ = 1.501 Mg m^−^^3^
Monoclinic, *P*2_1_/*c*	Cu *K*α radiation, λ = 1.54178 Å
*a* = 14.8117 (18) Å	Cell parameters from 9966 reflections
*b* = 10.7086 (13) Å	θ = 3.2–74.6°
*c* = 15.1887 (19) Å	µ = 3.80 mm^−^^1^
β = 112.417 (3)°	*T* = 150 K
*V* = 2227.1 (5) Å^3^	Plate, pale yellow
*Z* = 4	0.21 × 0.18 × 0.08 mm

### Data collection

**Table:**

Bruker D8 VENTURE PHOTON 100 CMOS diffractometer	4497 independent reflections
Radiation source: INCOATEC IµS micro–focus source	4000 reflections with *I* > 2σ(*I*)
Mirror monochromator	*R*_int_ = 0.031
Detector resolution: 10.4167 pixels mm^-1^	θ_max_ = 74.6°, θ_min_ = 3.2°
ω scans	*h* = −18→17
Absorption correction: numerical (*SADABS*; Krause *et al.*, 2015)	*k* = −13→12
*T*_min_ = 0.59, *T*_max_ = 0.76	*l* = −18→18
16958 measured reflections	

### Refinement

**Table:**

Refinement on *F*^2^	Primary atom site location: structure-invariant direct methods
Least-squares matrix: full	Secondary atom site location: difference Fourier map
*R*[*F*^2^ > 2σ(*F*^2^)] = 0.032	Hydrogen site location: mixed
*wR*(*F*^2^) = 0.082	H atoms treated by a mixture of independent and constrained refinement
*S* = 1.05	*w* = 1/[σ^2^(*F*_o_^2^) + (0.0409*P*)^2^ + 1.0281*P*] where *P* = (*F*_o_^2^ + 2*F*_c_^2^)/3
4497 reflections	(Δ/σ)_max_ = 0.001
367 parameters	Δρ_max_ = 0.23 e Å^−^^3^
0 restraints	Δρ_min_ = −0.35 e Å^−^^3^

### Special details

**Table:**

Geometry. All esds (except the esd in the dihedral angle between two l.s. planes) are estimated using the full covariance matrix. The cell esds are taken into account individually in the estimation of esds in distances, angles and torsion angles; correlations between esds in cell parameters are only used when they are defined by crystal symmetry. An approximate (isotropic) treatment of cell esds is used for estimating esds involving l.s. planes.
Refinement. Refinement of F^2^ against ALL reflections. The weighted R-factor wR and goodness of fit S are based on F^2^, conventional R-factors R are based on F, with F set to zero for negative F^2^. The threshold expression of F^2^ > 2sigma(F^2^) is used only for calculating R-factors(gt) etc. and is not relevant to the choice of reflections for refinement. R-factors based on F^2^ are statistically about twice as large as those based on F, and R- factors based on ALL data will be even larger. The hydrogen atoms attached to C10 were included as riding contributions in idealized positions since independent refinement of them led to an unsatisfactory geometry for this methyl group.

### Fractional atomic coordinates and isotropic or equivalent isotropic displacement parameters (Å^2^)

**Table:**

	*x*	*y*	*z*	*U*_iso_*/*U*_eq_	
Cl1	0.17142 (3)	0.13315 (4)	0.78029 (3)	0.03577 (12)	
Cl2	0.88333 (3)	−0.06239 (4)	0.59045 (3)	0.03541 (12)	
S1	0.54003 (3)	0.68437 (3)	0.77522 (3)	0.02275 (10)	
O1	0.06386 (8)	0.72960 (11)	0.49406 (8)	0.0282 (3)	
O2	0.12796 (7)	0.58575 (10)	0.42622 (7)	0.0214 (2)	
O3	0.46040 (8)	0.25187 (11)	0.44901 (8)	0.0277 (3)	
N1	0.35416 (9)	0.75804 (11)	0.71116 (9)	0.0191 (3)	
N2	0.39614 (8)	0.58809 (11)	0.63452 (9)	0.0167 (2)	
N3	0.45875 (9)	0.42363 (11)	0.57852 (9)	0.0182 (2)	
N4	0.54188 (9)	0.35323 (11)	0.58917 (9)	0.0185 (3)	
H4	0.5851 (16)	0.339 (2)	0.6478 (16)	0.034 (5)*	
C1	0.29435 (10)	0.54883 (13)	0.58144 (10)	0.0164 (3)	
H1	0.2883 (13)	0.5261 (17)	0.5176 (13)	0.020 (4)*	
C2	0.22891 (10)	0.66068 (13)	0.57591 (10)	0.0174 (3)	
C3	0.25829 (11)	0.75263 (14)	0.64191 (10)	0.0187 (3)	
C4	0.41675 (10)	0.68010 (13)	0.70116 (10)	0.0173 (3)	
C5	0.56960 (11)	0.55753 (14)	0.71172 (12)	0.0222 (3)	
H5A	0.6126 (14)	0.5860 (18)	0.6802 (14)	0.026 (5)*	
H5B	0.6020 (14)	0.490 (2)	0.7563 (14)	0.030 (5)*	
C6	0.47481 (10)	0.51442 (13)	0.63687 (10)	0.0171 (3)	
C7	0.13182 (10)	0.66442 (14)	0.49758 (10)	0.0187 (3)	
C8	0.03585 (11)	0.58383 (16)	0.34410 (11)	0.0242 (3)	
H8A	0.0046 (14)	0.6678 (18)	0.3345 (13)	0.025 (5)*	
H8B	0.0558 (14)	0.5674 (18)	0.2876 (14)	0.031 (5)*	
C9	−0.02993 (13)	0.48441 (18)	0.35603 (13)	0.0312 (4)	
H9A	−0.0462 (16)	0.505 (2)	0.4146 (16)	0.046 (6)*	
H9B	−0.0915 (16)	0.481 (2)	0.2994 (15)	0.036 (5)*	
H9C	0.0007 (15)	0.402 (2)	0.3634 (15)	0.036 (5)*	
C10	0.19597 (11)	0.85945 (15)	0.64967 (12)	0.0244 (3)	
H10A	0.163647	0.898217	0.586985	0.037*	
H10B	0.237071	0.921446	0.694614	0.037*	
H10C	0.146532	0.828365	0.672479	0.037*	
C11	0.26837 (10)	0.43898 (13)	0.63133 (11)	0.0173 (3)	
C12	0.20906 (11)	0.34314 (14)	0.57860 (11)	0.0225 (3)	
H12	0.1865 (15)	0.3439 (19)	0.5098 (15)	0.033 (5)*	
C13	0.17835 (12)	0.24801 (15)	0.62368 (12)	0.0257 (3)	
H13	0.1355 (16)	0.183 (2)	0.5870 (15)	0.038 (6)*	
C14	0.21023 (11)	0.24912 (14)	0.72190 (12)	0.0237 (3)	
C15	0.27240 (12)	0.34069 (15)	0.77649 (11)	0.0228 (3)	
H15	0.2958 (14)	0.3367 (18)	0.8437 (15)	0.026 (5)*	
C16	0.30021 (11)	0.43621 (14)	0.73052 (11)	0.0208 (3)	
H16	0.3430 (14)	0.5015 (19)	0.7694 (14)	0.029 (5)*	
C17	0.53321 (11)	0.26487 (14)	0.52138 (10)	0.0192 (3)	
C18	0.62243 (11)	0.18586 (14)	0.54149 (10)	0.0195 (3)	
C19	0.71484 (11)	0.22052 (15)	0.60544 (11)	0.0203 (3)	
H19	0.7245 (15)	0.2983 (19)	0.6372 (14)	0.030 (5)*	
C20	0.79527 (12)	0.14427 (15)	0.62055 (11)	0.0222 (3)	
H20	0.8601 (14)	0.1693 (18)	0.6609 (14)	0.025 (5)*	
C21	0.78269 (12)	0.03304 (15)	0.57072 (11)	0.0245 (3)	
C22	0.69176 (13)	−0.00299 (16)	0.50590 (12)	0.0274 (3)	
H22	0.6864 (15)	−0.080 (2)	0.4729 (15)	0.038 (6)*	
C23	0.61184 (12)	0.07413 (15)	0.49123 (11)	0.0245 (3)	
H23	0.5501 (15)	0.0493 (18)	0.4452 (15)	0.030 (5)*	

### Atomic displacement parameters (Å^2^)

**Table:**

	*U*^11^	*U*^22^	*U*^33^	*U*^12^	*U*^13^	*U*^23^
Cl1	0.0475 (3)	0.0231 (2)	0.0488 (3)	−0.00518 (16)	0.0318 (2)	0.00199 (17)
Cl2	0.0330 (2)	0.0397 (2)	0.0294 (2)	0.01712 (17)	0.00721 (18)	−0.00225 (17)
S1	0.01551 (18)	0.02385 (19)	0.02288 (19)	−0.00037 (13)	0.00060 (15)	−0.00508 (14)
O1	0.0180 (5)	0.0331 (6)	0.0285 (6)	0.0073 (4)	0.0032 (5)	−0.0054 (5)
O2	0.0141 (5)	0.0247 (5)	0.0197 (5)	0.0017 (4)	0.0002 (4)	−0.0039 (4)
O3	0.0221 (6)	0.0340 (6)	0.0211 (6)	0.0022 (5)	0.0016 (5)	−0.0051 (5)
N1	0.0173 (6)	0.0195 (6)	0.0184 (6)	−0.0006 (5)	0.0043 (5)	−0.0012 (5)
N2	0.0127 (6)	0.0167 (6)	0.0181 (6)	−0.0003 (4)	0.0030 (5)	−0.0006 (5)
N3	0.0155 (6)	0.0189 (6)	0.0192 (6)	0.0023 (5)	0.0053 (5)	0.0022 (5)
N4	0.0156 (6)	0.0200 (6)	0.0177 (6)	0.0037 (5)	0.0037 (5)	0.0006 (5)
C1	0.0124 (6)	0.0184 (7)	0.0155 (7)	−0.0002 (5)	0.0020 (5)	−0.0013 (5)
C2	0.0149 (7)	0.0178 (7)	0.0181 (7)	0.0008 (5)	0.0046 (6)	0.0012 (5)
C3	0.0174 (7)	0.0198 (7)	0.0180 (7)	0.0001 (5)	0.0058 (6)	0.0018 (5)
C4	0.0167 (7)	0.0171 (7)	0.0163 (7)	−0.0021 (5)	0.0044 (6)	0.0011 (5)
C5	0.0159 (7)	0.0218 (7)	0.0237 (8)	0.0003 (6)	0.0019 (6)	−0.0022 (6)
C6	0.0146 (6)	0.0171 (7)	0.0183 (7)	0.0005 (5)	0.0047 (6)	0.0038 (5)
C7	0.0170 (7)	0.0189 (7)	0.0187 (7)	−0.0001 (5)	0.0051 (6)	0.0008 (5)
C8	0.0169 (7)	0.0306 (8)	0.0187 (7)	0.0024 (6)	−0.0004 (6)	−0.0024 (6)
C9	0.0210 (8)	0.0382 (10)	0.0296 (9)	−0.0060 (7)	0.0042 (7)	−0.0050 (7)
C10	0.0213 (7)	0.0241 (8)	0.0245 (8)	0.0031 (6)	0.0050 (6)	−0.0044 (6)
C11	0.0137 (6)	0.0167 (7)	0.0208 (7)	0.0019 (5)	0.0056 (6)	0.0001 (5)
C12	0.0218 (7)	0.0212 (7)	0.0225 (8)	−0.0018 (6)	0.0063 (6)	−0.0025 (6)
C13	0.0248 (8)	0.0198 (7)	0.0323 (9)	−0.0051 (6)	0.0106 (7)	−0.0055 (6)
C14	0.0232 (8)	0.0183 (7)	0.0343 (9)	0.0008 (6)	0.0162 (7)	0.0017 (6)
C15	0.0242 (8)	0.0228 (7)	0.0220 (8)	0.0028 (6)	0.0096 (6)	0.0017 (6)
C16	0.0196 (7)	0.0196 (7)	0.0209 (7)	−0.0006 (6)	0.0052 (6)	−0.0012 (6)
C17	0.0196 (7)	0.0211 (7)	0.0166 (7)	−0.0011 (6)	0.0063 (6)	0.0017 (5)
C18	0.0207 (7)	0.0219 (7)	0.0162 (7)	0.0012 (6)	0.0076 (6)	0.0014 (5)
C19	0.0208 (7)	0.0217 (7)	0.0186 (7)	−0.0001 (6)	0.0078 (6)	0.0001 (6)
C20	0.0207 (7)	0.0259 (8)	0.0185 (7)	0.0011 (6)	0.0058 (6)	0.0029 (6)
C21	0.0267 (8)	0.0277 (8)	0.0199 (7)	0.0077 (6)	0.0097 (7)	0.0032 (6)
C22	0.0319 (9)	0.0262 (8)	0.0230 (8)	0.0042 (7)	0.0092 (7)	−0.0046 (6)
C23	0.0239 (8)	0.0266 (8)	0.0207 (8)	0.0003 (6)	0.0060 (7)	−0.0042 (6)

### Geometric parameters (Å, º)

**Table:**

Cl1—C14	1.7459 (16)	C8—H8B	1.02 (2)
Cl2—C21	1.7365 (16)	C9—H9A	1.03 (2)
S1—C4	1.7421 (15)	C9—H9B	0.99 (2)
S1—C5	1.8135 (16)	C9—H9C	0.98 (2)
O1—C7	1.2092 (18)	C10—H10A	0.9800
O2—C7	1.3566 (18)	C10—H10B	0.9800
O2—C8	1.4559 (18)	C10—H10C	0.9800
O3—C17	1.2193 (19)	C11—C12	1.389 (2)
N1—C4	1.2987 (19)	C11—C16	1.397 (2)
N1—C3	1.4097 (19)	C12—C13	1.397 (2)
N2—C4	1.3612 (19)	C12—H12	0.97 (2)
N2—C6	1.3963 (18)	C13—C14	1.382 (2)
N2—C1	1.4740 (17)	C13—H13	0.96 (2)
N3—C6	1.2754 (19)	C14—C15	1.384 (2)
N3—N4	1.3995 (17)	C15—C16	1.387 (2)
N4—C17	1.3680 (19)	C15—H15	0.95 (2)
N4—H4	0.89 (2)	C16—H16	0.98 (2)
C1—C2	1.5228 (19)	C17—C18	1.499 (2)
C1—C11	1.526 (2)	C18—C19	1.393 (2)
C1—H1	0.970 (18)	C18—C23	1.396 (2)
C2—C3	1.353 (2)	C19—C20	1.389 (2)
C2—C7	1.477 (2)	C19—H19	0.95 (2)
C3—C10	1.503 (2)	C20—C21	1.385 (2)
C5—C6	1.503 (2)	C20—H20	0.96 (2)
C5—H5A	0.98 (2)	C21—C22	1.386 (2)
C5—H5B	0.98 (2)	C22—C23	1.390 (2)
C8—C9	1.500 (2)	C22—H22	0.95 (2)
C8—H8A	1.00 (2)	C23—H23	0.95 (2)
			
C4—S1—C5	92.48 (7)	H9A—C9—H9C	110.0 (18)
C7—O2—C8	115.69 (11)	H9B—C9—H9C	107.6 (17)
C4—N1—C3	116.56 (12)	C3—C10—H10A	109.5
C4—N2—C6	116.21 (12)	C3—C10—H10B	109.5
C4—N2—C1	120.38 (12)	H10A—C10—H10B	109.5
C6—N2—C1	121.49 (12)	C3—C10—H10C	109.5
C6—N3—N4	114.05 (12)	H10A—C10—H10C	109.5
C17—N4—N3	117.37 (12)	H10B—C10—H10C	109.5
C17—N4—H4	117.6 (14)	C12—C11—C16	119.03 (14)
N3—N4—H4	118.2 (13)	C12—C11—C1	120.47 (13)
N2—C1—C2	107.61 (11)	C16—C11—C1	120.43 (13)
N2—C1—C11	110.33 (11)	C11—C12—C13	120.58 (15)
C2—C1—C11	110.98 (11)	C11—C12—H12	119.8 (12)
N2—C1—H1	107.7 (10)	C13—C12—H12	119.5 (12)
C2—C1—H1	109.1 (11)	C14—C13—C12	118.79 (15)
C11—C1—H1	111.0 (11)	C14—C13—H13	120.6 (13)
C3—C2—C7	121.09 (13)	C12—C13—H13	120.6 (13)
C3—C2—C1	120.89 (13)	C13—C14—C15	121.88 (15)
C7—C2—C1	118.01 (12)	C13—C14—Cl1	119.93 (12)
C2—C3—N1	122.05 (13)	C15—C14—Cl1	118.19 (13)
C2—C3—C10	125.34 (14)	C14—C15—C16	118.59 (15)
N1—C3—C10	112.60 (13)	C14—C15—H15	120.0 (12)
N1—C4—N2	125.77 (13)	C16—C15—H15	121.4 (12)
N1—C4—S1	121.79 (11)	C15—C16—C11	121.05 (14)
N2—C4—S1	112.44 (10)	C15—C16—H16	118.2 (12)
C6—C5—S1	106.67 (10)	C11—C16—H16	120.7 (12)
C6—C5—H5A	108.6 (11)	O3—C17—N4	123.67 (14)
S1—C5—H5A	111.1 (11)	O3—C17—C18	121.81 (14)
C6—C5—H5B	111.6 (12)	N4—C17—C18	114.50 (13)
S1—C5—H5B	109.7 (12)	C19—C18—C23	119.04 (14)
H5A—C5—H5B	109.2 (16)	C19—C18—C17	123.16 (14)
N3—C6—N2	118.70 (13)	C23—C18—C17	117.77 (14)
N3—C6—C5	129.25 (13)	C20—C19—C18	120.74 (15)
N2—C6—C5	112.05 (12)	C20—C19—H19	118.7 (12)
O1—C7—O2	122.84 (13)	C18—C19—H19	120.5 (12)
O1—C7—C2	126.15 (14)	C21—C20—C19	119.14 (15)
O2—C7—C2	110.99 (12)	C21—C20—H20	118.8 (11)
O2—C8—C9	110.25 (13)	C19—C20—H20	121.9 (11)
O2—C8—H8A	110.1 (11)	C20—C21—C22	121.28 (15)
C9—C8—H8A	111.8 (11)	C20—C21—Cl2	118.95 (13)
O2—C8—H8B	104.2 (11)	C22—C21—Cl2	119.77 (13)
C9—C8—H8B	112.8 (11)	C21—C22—C23	119.08 (15)
H8A—C8—H8B	107.4 (16)	C21—C22—H22	118.6 (13)
C8—C9—H9A	109.4 (13)	C23—C22—H22	122.3 (13)
C8—C9—H9B	110.2 (12)	C22—C23—C18	120.70 (15)
H9A—C9—H9B	108.5 (17)	C22—C23—H23	118.2 (12)
C8—C9—H9C	111.2 (12)	C18—C23—H23	121.1 (12)
			
C6—N3—N4—C17	−171.54 (13)	C1—C2—C7—O1	162.79 (15)
C4—N2—C1—C2	28.34 (17)	C3—C2—C7—O2	162.47 (13)
C6—N2—C1—C2	−168.05 (12)	C1—C2—C7—O2	−18.75 (18)
C4—N2—C1—C11	−92.88 (15)	C7—O2—C8—C9	−91.73 (16)
C6—N2—C1—C11	70.74 (16)	N2—C1—C11—C12	−142.35 (14)
N2—C1—C2—C3	−25.06 (18)	C2—C1—C11—C12	98.47 (16)
C11—C1—C2—C3	95.74 (16)	N2—C1—C11—C16	40.59 (18)
N2—C1—C2—C7	156.14 (12)	C2—C1—C11—C16	−78.60 (16)
C11—C1—C2—C7	−83.05 (16)	C16—C11—C12—C13	2.6 (2)
C7—C2—C3—N1	−173.09 (13)	C1—C11—C12—C13	−174.50 (14)
C1—C2—C3—N1	8.2 (2)	C11—C12—C13—C14	−1.8 (2)
C7—C2—C3—C10	5.8 (2)	C12—C13—C14—C15	−0.8 (2)
C1—C2—C3—C10	−172.92 (14)	C12—C13—C14—Cl1	178.85 (12)
C4—N1—C3—C2	8.7 (2)	C13—C14—C15—C16	2.4 (2)
C4—N1—C3—C10	−170.30 (13)	Cl1—C14—C15—C16	−177.23 (12)
C3—N1—C4—N2	−5.4 (2)	C14—C15—C16—C11	−1.5 (2)
C3—N1—C4—S1	174.58 (10)	C12—C11—C16—C15	−0.9 (2)
C6—N2—C4—N1	−179.75 (14)	C1—C11—C16—C15	176.17 (13)
C1—N2—C4—N1	−15.3 (2)	N3—N4—C17—O3	6.5 (2)
C6—N2—C4—S1	0.26 (16)	N3—N4—C17—C18	−175.14 (12)
C1—N2—C4—S1	164.71 (10)	O3—C17—C18—C19	159.59 (15)
C5—S1—C4—N1	−178.09 (13)	N4—C17—C18—C19	−18.8 (2)
C5—S1—C4—N2	1.90 (11)	O3—C17—C18—C23	−18.5 (2)
C4—S1—C5—C6	−3.28 (11)	N4—C17—C18—C23	163.08 (13)
N4—N3—C6—N2	−177.82 (12)	C23—C18—C19—C20	−1.2 (2)
N4—N3—C6—C5	2.2 (2)	C17—C18—C19—C20	−179.26 (14)
C4—N2—C6—N3	177.12 (13)	C18—C19—C20—C21	0.3 (2)
C1—N2—C6—N3	12.9 (2)	C19—C20—C21—C22	0.4 (2)
C4—N2—C6—C5	−2.92 (18)	C19—C20—C21—Cl2	−179.59 (12)
C1—N2—C6—C5	−167.18 (13)	C20—C21—C22—C23	−0.3 (2)
S1—C5—C6—N3	−176.04 (13)	Cl2—C21—C22—C23	179.70 (13)
S1—C5—C6—N2	4.01 (15)	C21—C22—C23—C18	−0.6 (2)
C8—O2—C7—O1	−0.2 (2)	C19—C18—C23—C22	1.3 (2)
C8—O2—C7—C2	−178.77 (12)	C17—C18—C23—C22	179.48 (14)
C3—C2—C7—O1	−16.0 (2)		

### Hydrogen-bond geometry (Å, º)

**Table:**

*D*—H···*A*	*D*—H	H···*A*	*D*···*A*	*D*—H···*A*
N4—H4···N1^i^	0.89 (2)	2.16 (2)	3.0076 (18)	158 (2)
C5—H5*A*···O3^ii^	0.98 (2)	2.533 (19)	3.081 (2)	115.3 (14)
C5—H5*B*···N1^i^	0.98 (2)	2.57 (2)	3.453 (2)	150.1 (16)
C8—H8*B*···Cl1^iii^	1.02 (2)	2.77 (2)	3.4430 (17)	123.1 (14)
C15—H15···O3^iv^	0.95 (2)	2.54 (2)	3.1682 (19)	124.5 (17)

Symmetry codes: (i) −*x*+1, *y*−1/2, −*z*+3/2; (ii) −*x*+1, −*y*+1, −*z*+1; (iii) *x*, −*y*+1/2, *z*−1/2; (iv) *x*, −*y*+1/2, *z*+1/2.
